# Angioid Streaks in Pseudoxanthoma Elasticum

**DOI:** 10.7759/cureus.15720

**Published:** 2021-06-17

**Authors:** Rahaf A Mandura, Rwan E Radi

**Affiliations:** 1 Department of Ophthalmology, King Abdul-Aziz University, Jeddah, SAU; 2 College of Medicine, Umm Al-Qura University, Mecca, SAU

**Keywords:** pseudoxanthoma elasticum, angioid streaks, visual acuity, autosomal dominant, autosomal recessive, ophthalmology, genetics

## Abstract

Pseudoxanthoma elasticum, or Gronblad-Strandberg syndrome, is an inherited disorder that involves multiple organ systems. The characteristic degeneration and calcification of the elastic fibers caused by this disease were first observed by Ferdinand Jean Darrier in 1896. We report a case of a 27-year-old female who was diagnosed with pseudoxanthoma elasticum based on a skin biopsy prior to her presentation to our ophthalmology outpatient clinic. The past ocular history of the patient was unremarkable for any previous eye complaint or surgery. Her ocular and fundus examination showed pigmented grayish irregular post choroidal crack-like linear dehiscence, forming a network-like pattern, originating at the optic disc and extending radially involving the macular area and the posterior pole in both eyes, representing bilateral angioid streaks. There were no clinical or optical coherent tomographic signs of choroidal neovascularization. Periodic follow up for patients with pseudoxanthoma elasticum is recommended to detect choroidal neovascularization which is a sight-threatening complication. Ophthalmologists should be aware of this association as early recognition and treatment are vital to prevent irreversible visual loss.

## Introduction

Pseudoxanthoma elasticum (PXE), or Gronblad-Strandberg syndrome, is an inherited disorder that involves multiple organ systems. The hallmark of this disease is the mineralization and fragmentation of the elastic fibers in different tissues, which was first observed and described by Ferdinand Jean Darrier in 1896 [1]. It can be either inherited as autosomal dominant (AD) or autosomal recessive (AR) with each variant of inheritance having two subcategories, type 1 and type 2 [1]. AD type 1 disease is characterized by a rash among the flexor surfaces, angina provoked by effort, elevated blood pressure, and severe chorioretinitis. On the other hand, AD type 2 disease is considered milder and is characterized by a macular rash with no vascular changes while its ocular manifestation includes mild retinal degeneration [2,3]. The incidence of PXE is 1:1,600,000 individuals [4]. It affects females more than male with a female to male ratio of 2:1 and a mean onset of the disease around 13 years [4].

Clinically, three organ systems are mainly affected by PXE: the eyes, skin and cardiovascular system [4]. The skin is the furthermost and is usually the first organ affected by this disease [1]. The clinical findings of PXE usually become apparent when the affected individuals reach the second and third decade of life [5]. The ocular characteristic finding of this disease is called angioid streaks. They are often bilateral and consist of visible, irregular, linear, crack-like dehiscence of the calcified and brittle Bruch membrane. Early-onset of angioid streaks is proven to be associated with alteration in p.R1268Q [6,7]. This is a case report of a patient presenting with angioid streaks reported from King Abdul-Aziz University Hospital, Jeddah, Saudi Arabia.

## Case presentation

A 27-year-old female patient was diagnosed with PXE based on a skin biopsy a few weeks prior to her presentation. She presented to our ophthalmology outpatient clinic for routine screening. Past ocular history was unremarkable for any previous eye complaint or surgery. Past medical history was positive for long-standing skin lesions in the neck and axilla for seven years and the patient was followed at the dermatology clinic. On examination of the neck and the axilla, there were frontal and back hyperpigmented patches and atrophic spots.

**Figure 1:**
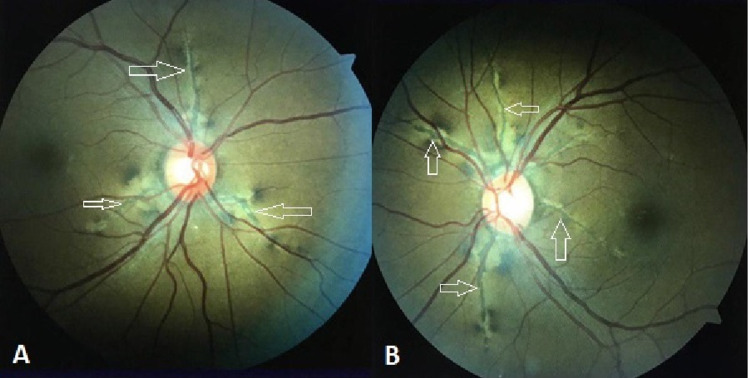
Fundus showing bilateral angioid streaks in the posterior pole (A) Right eye. (B) Left eye. White arrows: angioid streaks

Detailed ophthalmic examination of the patient showed the best-corrected visual acuity was 20/20 in the right eye (OD) and 20/20 in the left eye (OS). Intraocular pressure measured by a non-contact tonometer was 20 mmHg OD and 21 mmHg OS. The anterior segment examination was unremarkable in both eyes. Eye alignment by Hirschberg test and cover-uncover test showed orthotropic alignment in both eyes. Extraocular muscle movement showed a full range of movement without limitation bilaterally. Dilated fundus examination showed bilateral pigmented grayish irregular post choroidal crack-like linear dehiscence, forming a network-like pattern, originating at the optic disc from all sides and extending radially involving the macular area and the posterior pole in both eyes. The area surrounding these lesions showed degeneration of retinal pigment epithelium (RPE). These jagged lines were definite angioid streaks in the peripapillary area were radiating outward from the disc margins underlying the retinal blood vessels and not extending beyond the equator. There was no sign of choroidal neovascularization (CNV) or retinal hemorrhages. Normal fovea, flat retina, and clear vitreous were observed without signs of chorioretinitis or vitritis were noted (Figures 1A, 1B). Once the diagnosis was established, no treatment was needed at this time, and the patient was advised to attend her regular ophthalmology outpatient appointments to detect any progression or complication of her ocular condition. In addition, she was counselled regarding the high susceptibility of choroidal rupture and subretinal bleeding in patients with angioid streaks after minor blunt ocular trauma and encouraged to wear rigid eye protection especially during participation in contact sports.

## Discussion

Angioid streaks appear as a network that branches from the disc extending outwards into the retinal peripheries with borders of variable width. It appears with black or brown discoloration where they could be mistaken with retinal vessels. Retinal vessels extend as gray streaks surrounded with white lines and they are more superficial in comparison to angioid streaks, which are situated more deeply in the retina [3]. It was first observed and described by Deschweinitz in 1896, as a hereditary eye disease that appears mostly in brothers, siblings, and individuals of the same family [8-10]. It becomes clinically recognizable mostly when affected individuals reach their second until the fifth decade of life. Its presentation also depends on the underlying systemic disease of the patients and other affected organ systems [3,11]. Its incidence in patients with PXE is estimated to be 59% if the disease is diagnosed clinically and 87% if the disease is diagnosed by skin biopsy. It would be present in all patients with PXE about 20 years after diagnosis, and PXE is considered the most common disease associated with it [12].

Bruch membrane is rich in elastin and collagen and functions as a transporter of important nutrients and metabolites between the choriocapillaris and RPE [13]. The mineralization of this membrane due to PXE is the underlying pathogenesis of angioid streaks. In PXE, the lack of anti-mineralization factor leads to calcium deposition in all elastic tissues all over the body. This calcification of the elastin-rich Bruch membrane leads to rupture of the blood vessels with subsequent deterioration of vision clinically [14].

On dividing the pathogenesis of angioid streaks into stages, the first stage is thickening in the Bruch membrane, this thickening is associated with or without a decrease of the pigment granules, followed by pigment stripping, and mottling or formation of pigment clumps in the RPE. This pathway leads to CNV, which is the cause of the deterioration of vision [15]. The pathognomonic finding of PXE in the fundoscopic examination is mottled fundus or what is described by (peau d’orange) [16], their color is usually gray, red, brown, or mixed. They mostly localized to the peripapillary region and extended out to the posterior pole. If the location of the lesion is away from the fovea, the patient may have completely normal vision. However, lesions involving the fovea are associated with poor vision [17,18].

Treatment of angioid streaks depends on multiple factors including most importantly the location of the lesions near to or away from the fovea and whether the patient developed CNV or not yet. Intervention is preserved for patients who developed CNV, and the most recent treatment is anti-vascular endothelial growth factor injections such as ranibizumab, aflibercept, and off-label bevacizumab that have shown to be effective in stabilizing the visual acuity [19]. Our patient has good visual acuity and no development of CNV was noted; therefore, she was advised to attend the regular follow up visits to monitor her ocular findings.

## Conclusions

PXE is an inherited disorder that involves multiple organ systems including the eyes, skin, and cardiovascular system. The associated ocular finding is called angioid streaks, which are often bilateral and appear as irregular, linear, crack-like dehiscence of a calcified and brittle Bruch membrane. Periodic follow up for those patients is recommended to detect the sight-threatening complication, which is CNV, along with the education about wearing eye protection as they are at a higher risk of choroidal rupture even with minor blunt trauma. Ophthalmologists should be aware of these associations as early recognition and treatment are vital to prevent irreversible visual loss.
